# Satellite remote sensing enables monitoring of soil organic carbon decline in croplands of Jilin China

**DOI:** 10.1038/s41598-026-38386-x

**Published:** 2026-02-02

**Authors:** Zhengyuan Xu, De Hou, Nan Lin, Wenhao Liu, Tianyi Dong, Hao Chen, Zhongxu Wang

**Affiliations:** 1 School of Modern Industry, Jilin Jianzhu University, Changchun, 130118 China; 2 College of Surveying and Exploration Engineering, Jilin Jianzhu University, Changchun, 130118 China; 3 Heilongjiang Provincial Research Center of Geological and Mineral Experimental Testing, Harbin, 150036 China; 4 College of Geo-Exploration Science and Technology, Jilin University, Changchun, 130026 China

**Keywords:** Soil organic carbon (SOC), SOC decline, Broadband spectral index, Jilin province, Multi-source remote sensing, Agroecology, Environmental impact

## Abstract

Soil organic carbon (SOC) is a key parameter for soil quality. As one of the major grain-producing regions of China, Jilin Province plays a critical role in ensuring national food security, making cropland SOC monitoring essential. Based on satellite remote sensing observations, this study reveals an overall 5.14% decline in SOC across croplands in Jilin Province over the past seven years. Losses were most pronounced in the west, while the central and eastern areas remained relatively stable. Conventional SOC estimation methods largely rely on machine learning, which can lack physical interpretability and reproducibility. PLSR-based SOC models achieved validation R^2^ values of 0.40–0.61 with corresponding RMSEs of 0.30–0.38 across MODIS Terra, Landsat OLI, and Sentinel-2 MSI. The quantitative models exhibit satisfactory validation accuracy but limited spatial robustness across sensors in practical mapping. This study proposes a new broadband spectral index, the Ratio Soil Index (RSI), applied at 30-meter resolution. Using field synchronized SOC measurements and spectral analysis, we developed broadband indices from MODIS Terra, Landsat OLI, and Sentinel MSI. The RSI showed strong correlations with measured SOC, with coefficients of 0.72, 0.74, and 0.77 for the three sensors. Its spatial patterns were consistent with ground observations within the 95% confidence interval. The findings demonstrate that the RSI, with its concise formulation, reliable mapping performance, and ability to identify the variations of SOC, offers a scalable and reproducible metric for national SOC monitoring under changing agricultural management.

## Introduction

Soil constitutes a fundamental component of terrestrial ecosystems, playing a pivotal role in sustaining life through various ecosystem services, including biomass synthesis, water and nutrient storage and cycling, carbon sequestration, and habitat provision for biological activities^1^. Jilin Province, located in the core area of the black soil region in Northeast China, is a major agricultural province. However, its croplands are facing the challenge of topsoil degradation^2,3^. In response, China has been prioritizing the conservation of black soil regions^3^. Soil organic carbon (SOC) plays a crucial role in water and nutrient retention, nutrient utilization, and biodiversity maintenance. As a core component of soil function, it is recognized as a key indicator for assessing cropland soil quality^4^. Monitoring the spatiotemporal dynamics of SOC in croplands across Jilin Province under changing agricultural management is of great importance for modern agricultural production.

Conventional approaches for monitoring the spatial and temporal variations of SOC rely on localized sampling and laboratory-based chemical analysis, which are costly, time-consuming, and spatially sparse. Existing studies have reported historical SOC changes only in specific areas of Jilin Province^5^, and to date, there has been no comprehensive monitoring of spatiotemporal changes in SOC across the entire cropland area of Jilin Province. SOC estimation based on remote sensing observations offers rapid, large-scale, and timely monitoring, and has therefore increasingly leveraged multispectral sensors with complementary spatiotemporal characteristics. Sentinel-2 MSI provides red-edge and SWIR bands at 10–20 m resolution and has been demonstrated to support field-calibrated SOC retrieval and high-resolution SOC mapping over croplands, particularly under bare-soil or seasonally optimized conditions^6^. Landsat imagery offers a long, consistent archive at 30 m resolution, enabling SOC mapping and temporal mosaicking approaches for regional assessments and change analysis^7^. At broader spatial extents, MODIS observations offer high temporal frequency that supports large-area SOC monitoring through time-series compositing approaches, despite their coarser spatial resolution^8^. However, existing studies are often sensor-specific and model-dependent, which can limit cross-sensor comparability and reproducibility due to the inherent complexity of soils and heterogeneous spectral responses across satellite sensors. Within soils, chromophores such as clay minerals, water, organic carbon, iron oxides, and carbonates exhibit characteristic spectral characteristics in the visible-near infrared to shortwave infrared range^9^. Given the inherent complexity of soil as a system, the transfer of electromagnetic waves within this heterogeneous medium exhibits significant uncertainty. Consequently, quantitatively estimating soil properties from spectra remains challenging^9,10^. Although active functional groups in soils, including C-H, C-N, C=O, and -OH, and electronic transitions, give rise to characteristic spectral absorption features^11^, the influence of chromophores on soil spectral characteristics is neither simple nor linear, due to complex interactions among functional groups^12^. As a result, effectively predicting and inverting soil spectral characteristics using physical theoretical models becomes highly challenging^9,13,14^. Consequently, research on the quantitative remote sensing evaluation of SOC has increasingly adopted a calibration-validation strategy^9,15,16^. Multivariate linear models, including stepwise multiple linear regression (SMLR), principal component regression (PCR), partial least squares regression (PLSR), and boosted regression tree (BRT) analysis, have been demonstrated to effectively enable the quantitative analysis of SOC using proximal sensing hyperspectral data^17–19^. With advancements in machine learning techniques, methods such as artificial neural networks (ANN), random forests (RF), support vector machines (SVM), and k-nearest neighbor algorithms (KNN) have also been introduced to facilitate local-scale quantitative estimation of SOC content^20^. Among these approaches, PLSR has been proven to be one of the most effective algorithms for SOC estimation, owing to its ability to project high-dimensional collinear variables onto low-dimensional orthogonal components, thereby achieving dimensionality reduction^21^. However, the calibration-validation process has inherent limitations^22^. Specifically, variations in spectral characteristics caused by sample heterogeneity can lead to model failure^23^, while the complexity of machine learning model expressions often restricts model sharing and cross-application validation^24^.

In the 1970s, the Great Plains Corridor Rangeland Project carried out statistical analysis on green vegetation biomass data collected over a one-year period from 10 ground stations across six states in the Great Plains. These data were analyzed with synchronously acquired ERTS-1 MSS imagery, revealing a strong correlation between the spectral difference in the red and near-infrared bands and green vegetation biomass, leading to the development of the Normalized Difference Vegetation Index (NDVI). This spectral index remains one of the most widely used indicators for quantitatively assessing vegetation growth^25^. Remote sensing-based vegetation monitoring has since advanced significantly, giving rise to numerous broadband vegetation indices, such as the ratio vegetation index (RVI)^26^, the difference vegetation index (DVI)^27^, and the later-developed wide dynamic range vegetation index (WDRVI)^28^. With the ongoing advancement of spectral index research, increasing attention has been paid within diverse research fields to the use of band combinations in remote sensing data for quantitatively assessing surface target properties. This approach led to the development of remote sensing-based indices for specific applications, such as the Normalized Difference Water Index (NDWI) for surface water extraction and analysis^29^, and the Temperature Vegetation Dryness Index (TVDI) for surface drought monitoring^30^. These spectral indices have been widely validated and applied in environmental monitoring, establishing spectral index-based remote sensing as an effective and efficient method for large-scale, high-temporal-resolution quantitative assessment of surface features. However, compared to the broad applications of vegetation, water, and drought indices, soil has often been treated as a background disturbance rather than as a primary subject of quantitative remote sensing evaluation^31^. To date, no universally recognized and widely adopted spectral index for soil monitoring has been proposed and promoted in the scientific community. Some studies have attempted to explore the relationship between two-band combinations and SOC content based on laboratory-acquired soil hyperspectral data. For example, using laboratory spectral data from ten soil samples collected in Illinois, USA, SOC content estimation models have been developed based on three two-band spectral combination factors, with sensitive bands concentrated around the 600 nm spectral region^32^. Jin et al.(2016) provided a review of studies on SOC-sensitive band combinations derived from laboratory spectral data and conducted replication experiments based on 237 samples^33^. However, these findings were derived from hyperspectral soil data obtained under controlled conditions. Currently, there remains a lack of research on the direct application of spectral indices to satellite remote sensing data for SOC quantitative evaluation.

We hypothesize that two-band broadband spectral indices can effectively estimate SOC, therefore, the objective of this study is to develop a spectral index that is sensitive to SOC and applicable at the provincial scale, enabling the tracking of the temporal decline of SOC in croplands across Jilin Province. Using the entire cropland area of Jilin Province, China, as the study area, we integrate multisource remote sensing data, including Landsat OLI, MODIS Terra, and Sentinel-2 MSI, with ground sampling and synchronized field observations to conduct spectral analysis and construct broadband spectral indices. On this basis, Landsat OLI time-series data are used to monitor changes in the spectral index, and SOC sample data from 2017 and 2024 are incorporated to characterize and validate the recent spatiotemporal variation of SOC across croplands in Jilin Province, China.

## Results

**Fig. 1:**
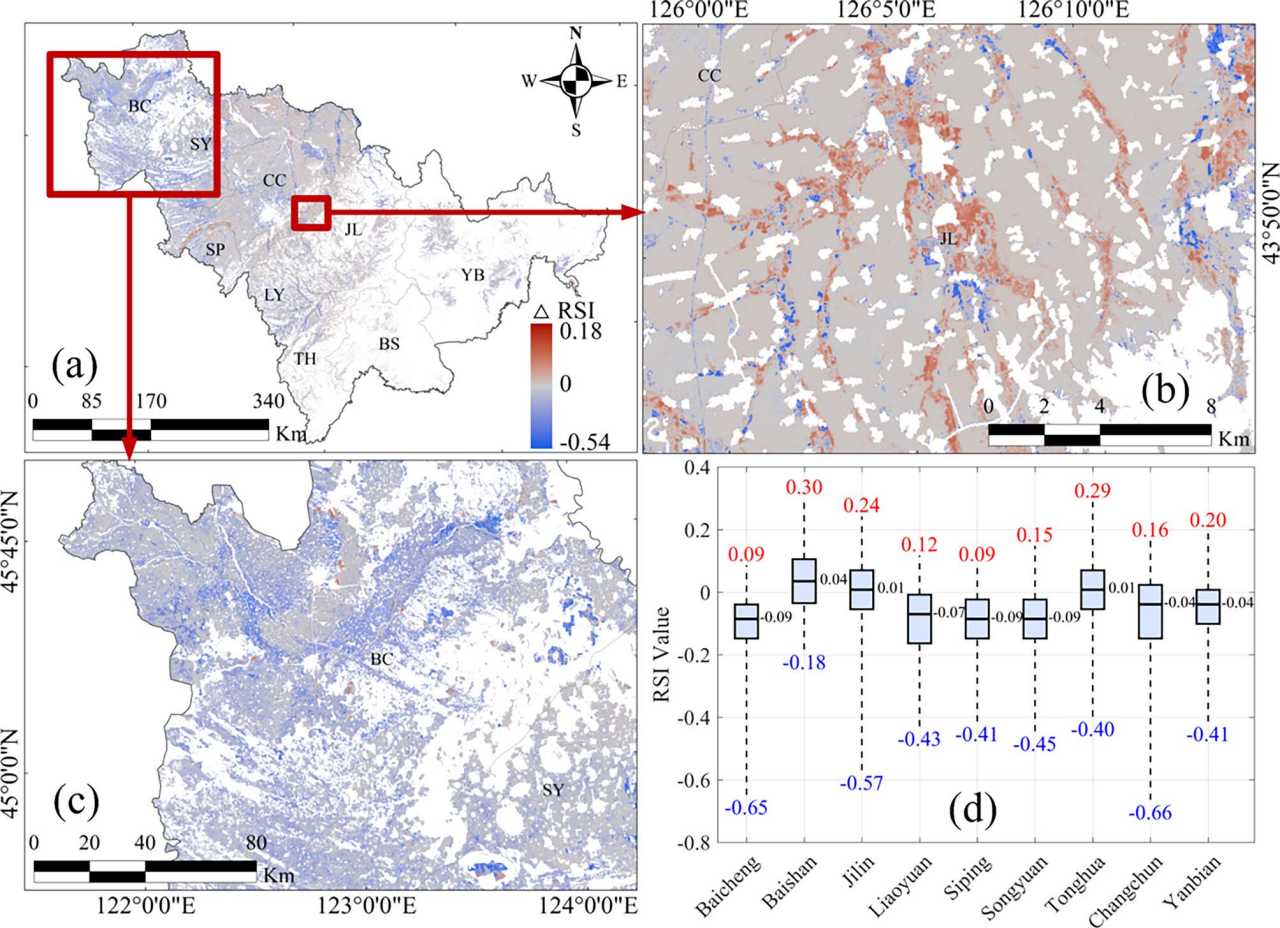
Changes in the RSI across croplands in Jilin Province from 2017 to 2024. (**a**) Overall spatial pattern of RSI changes in cropland areas across the province. (**b**) Localized increasing trend observed in parts of Jilin City. (**c)** General declining trend in the western regions. (**d**) Regional statistical summary of RSI changes. Notes: Landsat OLI imagery, 30 m spatial resolution; Coordinate system CGCS2000/3° Gauss–Krüger Zone 41. This figure was generated in ArcGIS 10.2 software (https://www.esri.com/zh-cn/home).

In the cropland regions of Jilin Province, the Ratio Soil Index (RSI) developed in this study based on Landsat OLI data reveals an overall declining trend in SOC content from 2017 to 2024(Fig. 1), which is consistent with recent related findings^34^. The provincial median RSI value decreased from 1.36 in 2017 to 1.29 in 2024, corresponding to an overall reduction of 5.14%. Across the entire study area, the maximum and minimum RSI changes (△RSI) were 0.18 and –0.54, respectively, based on statistics within the 95% confidence interval (Fig. 1a). At the regional scale, the most significant SOC decline occurred in the western part of the province, particularly in Baicheng and Songyuan, where RSI values decreased markedly (Fig. 1c). In Baicheng, the median RSI decreased from 1.38 to 1.29 (-6.22%), and in Songyuan, from 1.39 to 1.30 (-6.19%), based on zonal statistics. In contrast, the central black soil region exhibited only minor variation, with RSI values remaining generally stable. In Jilin City, RSI values showed an upward trend (Fig. 1b), with the median increasing from 1.24 to 1.25. Notably, a significant increase in SOC was also observed in Baishan. However, due to the limited extent of cropland and the small number of pixels involved in the regional statistics, the representativeness of this result may be limited. Spatially, RSI changes in the central region exhibit a banded pattern aligned with the Songhua River system, while croplands outside the floodplain show no significant variation. This spatial configuration indicates that SOC variation is strongly influenced by soil erosion processes associated with fluvial dynamics. A summary of regional RSI changes over the past seven years across different areas of Jilin Province is presented in Fig. 1d

**Fig. 2:**
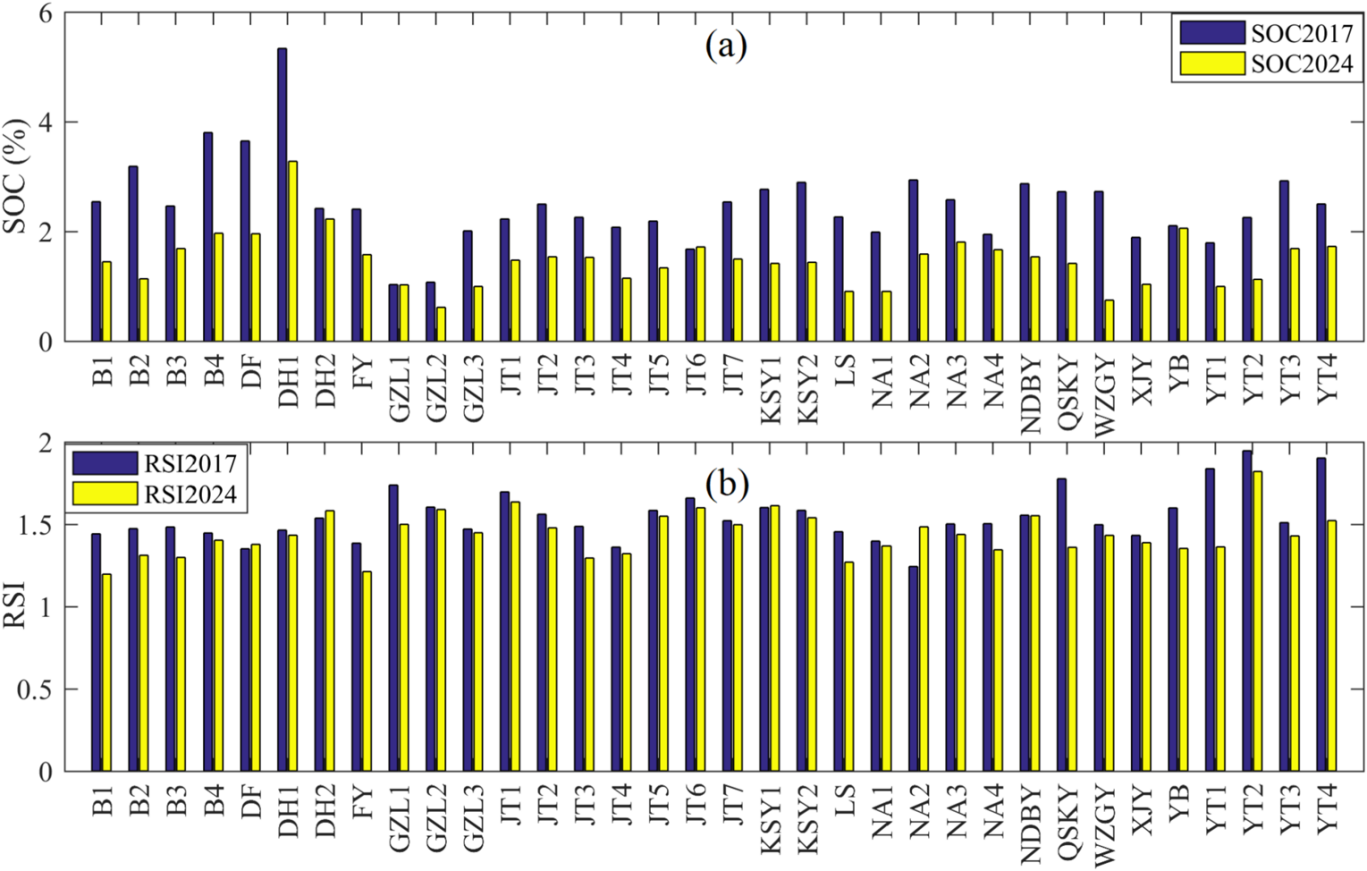
Changes in SOC and RSI at sample points from 2017 to 2024. (**a**) Changes in SOC content at individual sample sites. (**b**) Corresponding changes in RSI values at the same sample sites.

The SOC measurements from soil samples collected at field sampling sites between 2017 and 2024 clearly indicate an overall declining trend in SOC content (Fig. 2a). The corresponding RSI values extracted for these sample locations in 2017 and 2024 demonstrate that RSI generally responds well to the observed decline in SOC (Fig. 2b). However, at a few specific sites—such as the “DF” sampling point in Dongfeng, “DH2” in Dunhua, and “KSY1” and “NA2” in Changchun—the RSI values show an increasing trend, which contrasts with the decrease in SOC content measured in soil samples. This discrepancy between remotely sensed estimates and ground-based measurements is likely influenced by surface cover heterogeneity, which will be further discussed in a subsequent section.

### Modeling and validation

**Fig. 3:**
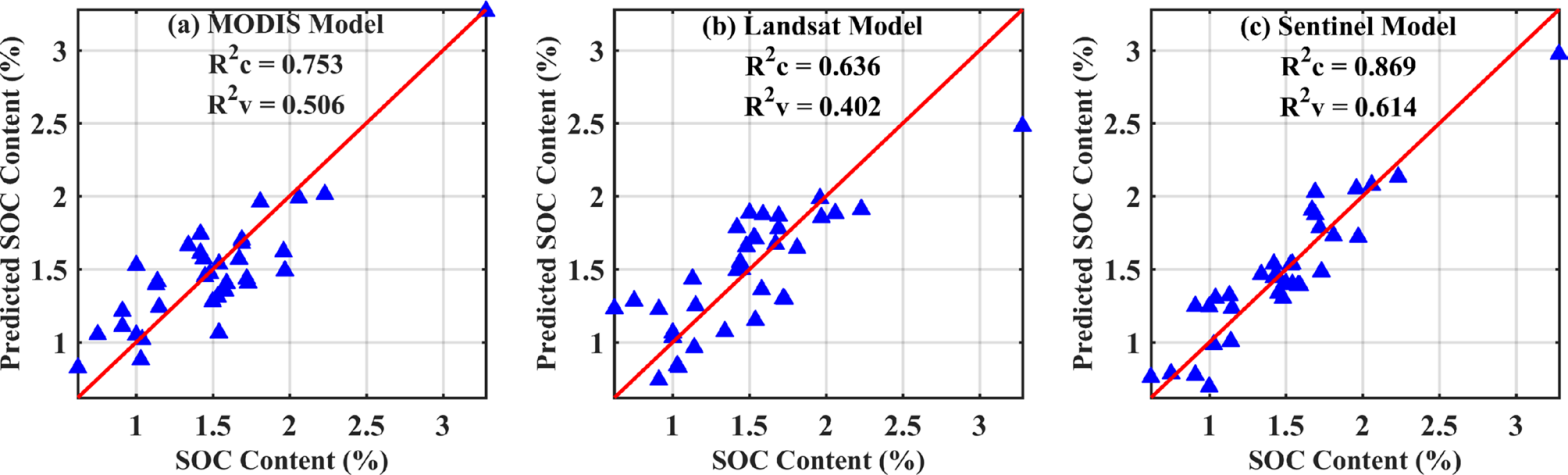
The calibration and validation results of the PLS models.

Spectral reflectance data from MODIS, Landsat, and Sentinel imagery were extracted for 34 sample sites, and a SOC inversion model was developed using the PLSR algorithm. The goodness-of-fit and error statistics for the three models are presented in Table 1. Fig. 3 illustrates the comparison between observed and estimated SOC content, along with model accuracy. Although MODIS data have a lower spatial resolution and suffer from spectral mixing effects more significantly than the other two datasets, the model still achieved relatively satisfactory accuracy. The Landsat-based model exhibited lower performance in both calibration and validation, whereas the Sentinel-2 MSI-based model yielded the highest accuracy, with R^2^c = 0.869 and R^2^v = 0.614.

**Table 1:** Goodness-of-fit and error metrics for predictive models.

**Models**	**R**^**2**^**c**	**RMSEc**	**R**^**2**^**v**	**RMSEv**
Model1[MODIS]	0.753	0.243	0.506	0.344
Model2[Landsat]	0.636	0.295	0.402	0.379
Model3[Sentinel]	0.869	0.177	0.614	0.304

Note: R^2^c: Coefficient of determination for calibration, RMSEc: Root mean squared error for calibration, R^2^v: Coefficient of determination for validation, RMSEv: Root mean squared error for validation.

### RSI development

**Fig. 4:**
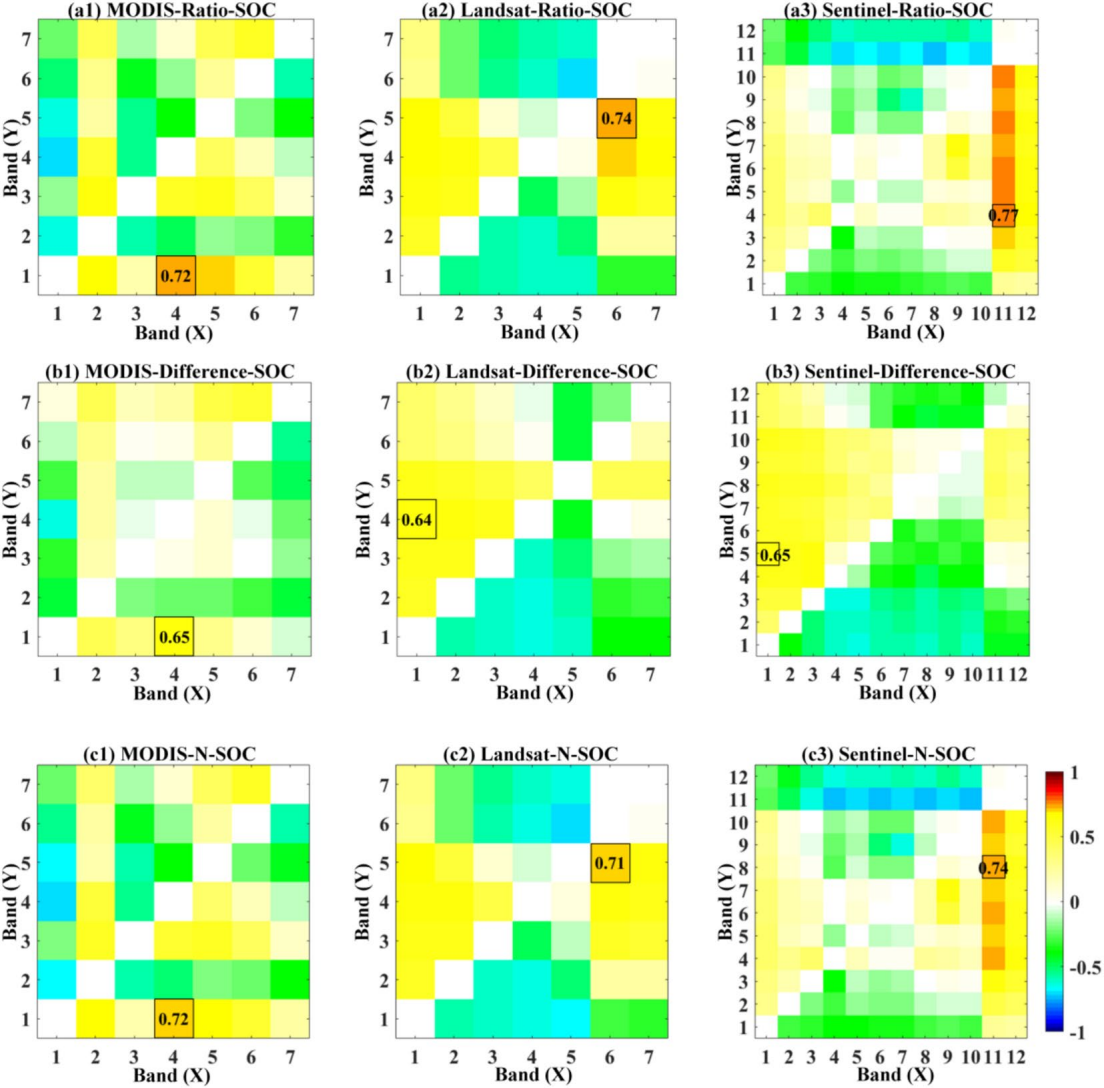
(**a1–a3**) Show the correlation coefficients between two-band ratio indices and SOC content for each of the three satellite datasets. Correspondingly, (**b1–b3**) show the correlation coefficients between two-band difference indices and SOC content. Similarly, (**c1–c3**) show the correlation coefficients between two-band normalized difference indices and SOC content. Axis X indicates the first band used in index computation. Axis Y indicates the second band used in index computation.

The spectral reflectance data from sampled sites were used to compute two-band ratio, difference, and normalized difference indices. By iterating through all spectral bands, various two-band combination spectral indices were generated, and their correlation coefficients with the measured SOC content were calculated. Fig. 4 presents the responses of different spectral indices to SOC across various band combinations and index formulations derived from the three satellite datasets. Table 2 lists the optimal results for these indices.

**Table 2:** Correlation coefficients between broadband soil spectral indices and SOC content.

**Index**	Correlation coefficient [Expression]		
	**MODIS**	**Landsat**	**Sentinel**
Ratio	0.72[b4/b1]	0.74[b6/b5]	0.77[b11/b4]
Difference	0.65[b4-b1]	0.64[b1-b4]	0.65[b1-b5]
Normalized difference	0.72[(b4-b1)/(b4+b1)]	0.71[(b6-b5)/(b6+b5)]	0.74[(b11-b8)/(b11+b8)]

It can be observed that the optimal indices from all three datasets exhibit a good response to SOC content. The difference indices shown in Fig. 4(b1)–(b3) are slightly less effective than the ratio indices in Fig. 4(a1)–(a3) and the normalized difference indices in Fig. 4(c1)–(c3). Among the three datasets, Sentinel-2 MSI-derived spectral indices demonstrate higher accuracy than those from the other two datasets, likely due to its higher spectral resolution. From an overall perspective, the MODIS data show that the spectral combination of b4 (Green) and b1 (Red) bands is most sensitive to SOC. The Landsat OLI and Sentinel MSI datasets yield similar results, with strong SOC response coefficients observed for spectral combinations involving the SWIR1 band and bands from the green to SWIR regions. The highest sensitivity is observed near the SWIR and NIR band combinations. Given that ratio indices achieve relatively high accuracy while maintaining a simple mathematical expression, the Ratio Soil Index (RSI) derived from all three satellite datasets will be selected for comparison with the PLSR inversion model in the following mapping analysis.

### SOC estimation and RSI mapping

**Fig. 5:**
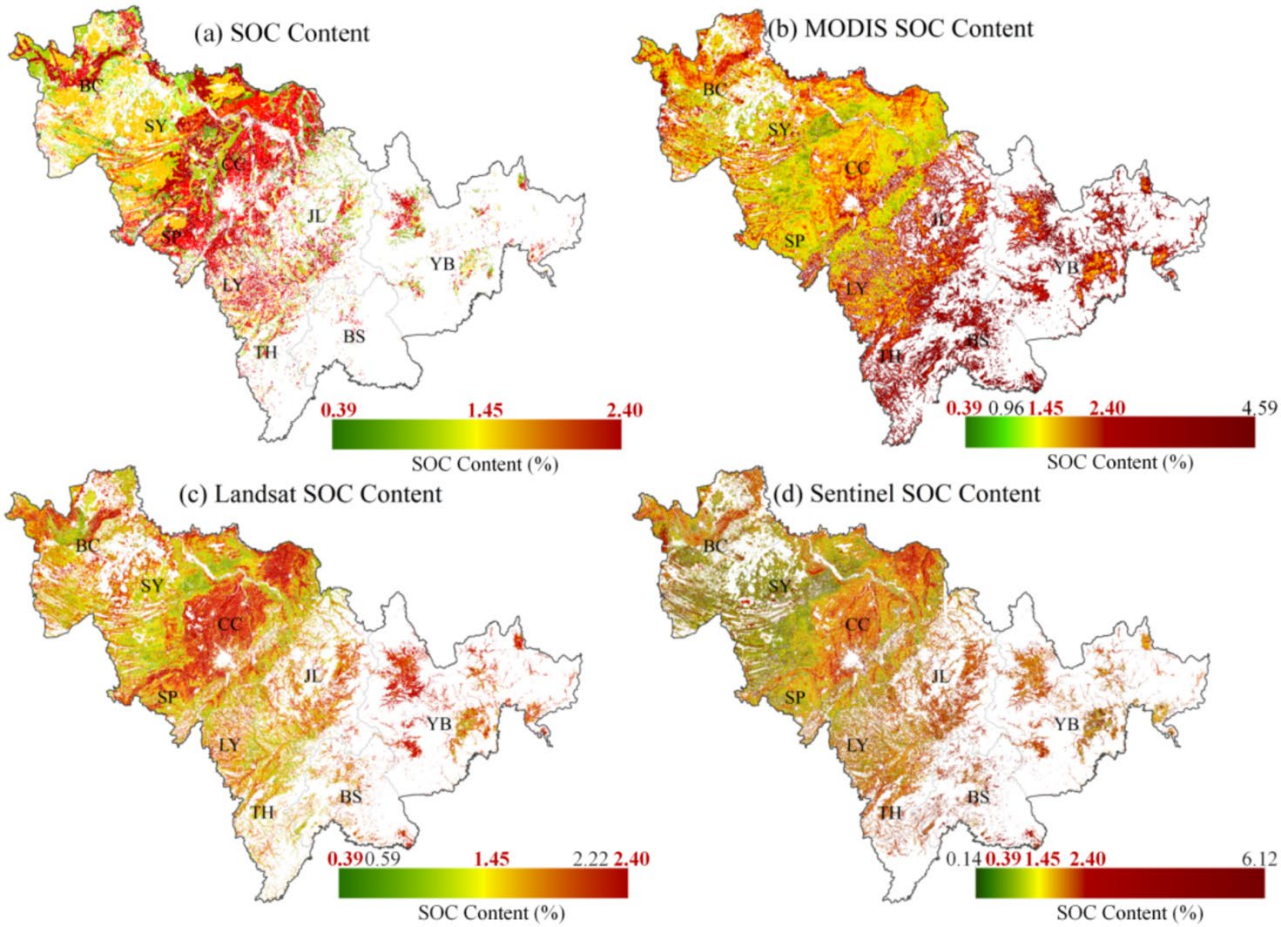
Mapping results. (**a**) The spatial distribution of observed SOC content in the farmlands of Jilin Province. (**b–d**) SOC inversion results obtained from PLSR models based on MODIS (200 m), Landsat OLI (30 m), and Sentinel-2 MSI (20 m) datasets. Notes: The observed SOC content is marked in red in the legend as a reference; Coordinate system CGCS2000/3° Gauss–Krüger Zone 41; The “Lower” and “Upper” of SOC content refer to the 95% confidence interval. This figure was generated in ArcGIS 10.2 software (https://www.esri.com/zh-cn/home).

Due to the limited number of collected samples, the discrete distribution of sampling points cannot fully represent the overall spatial characteristics of SOC in Jilin Province. Therefore, surface SOC data from the HWSD database were introduced as a standard reference, as shown in Fig. 5a. It is important to note that the HWSD dataset for China is based on historical sample collections, meaning the data do not reflect real-time absolute SOC content and are used only as a relative reference. The croplands of Jilin Province are primarily concentrated in the central and western plains, where the spatial distribution of SOC exhibits a general pattern of higher values in the central region, lower values in the west, and fragmented variations in the east. The central region lies within the typical black soil zone of Northeast China, where SOC content is noticeably lower than in the saline-alkali soils of northwestern Jilin. This difference is mainly attributed to the presence of Acrisols, which enhance SOC leaching processes in the central area. To ensure comparability between inversion results and measured SOC content, the color scales of the three inversion maps were standardized based on the SOC gradient. If an inversion result overestimates or underestimates SOC compared to the reference, the color scale is extended accordingly. In all inversion results, “Lower” and “Upper” SOC content refer to the 95% confidence interval. As shown in Fig. 5c,d, the Landsat and Sentinel-derived SOC inversion results successfully depict the overall spatial distribution pattern of SOC in Jilin Province. However, noticeable differences exist. The Landsat inversion closely aligns with the reference dataset, whereas the Sentinel-based inversion shows both underestimation and overestimation across different regions. Although the MODIS-based model demonstrated good validation accuracy, its actual inversion results exhibit substantial deviations from the reference, with a general overestimation of SOC.

**Fig. 6:**
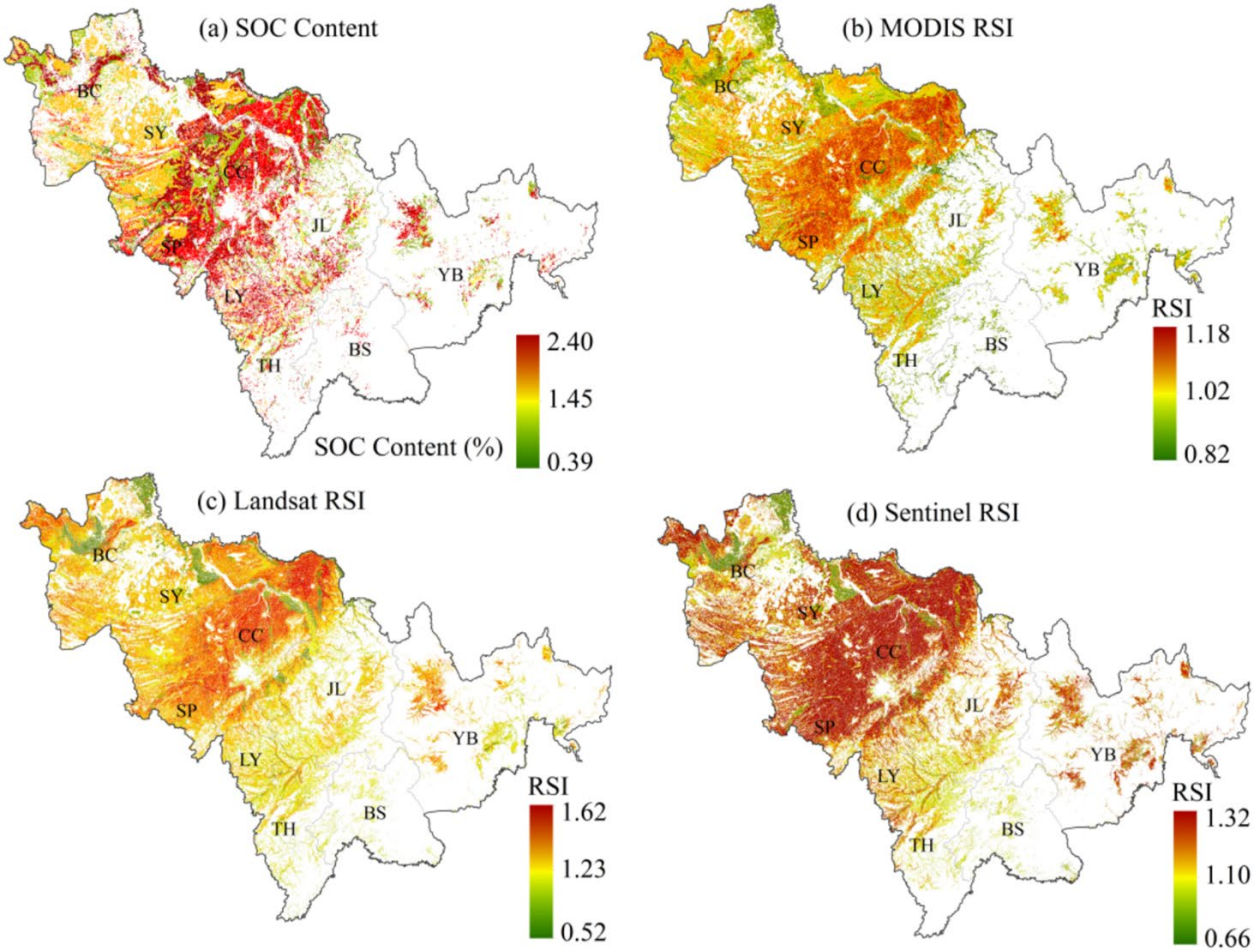
Mapping results. (**a**) The spatial distribution of observed SOC content in the farmlands of Jilin Province. (**b–d**) show the RSI calculation and mapping results derived from the MODIS (200 m), Landsat OLI (30 m), and Sentinel-2 MSI (20 m) datasets. Notes: Coordinate system CGCS2000/3° Gauss–Krüger Zone 41; The “Lower” and “Upper” of RSI refer to the 95% confidence interval. This figure was generated in ArcGIS 10.2 software (https://www.esri.com/zh-cn/home).

Similarly, using the measured SOC data from the HWSD dataset as a reference, the RSI maps derived from the three satellite datasets are presented in Fig. 6. The results indicate that RSI successfully captures the spatial distribution characteristics of SOC across the study area in all three satellite datasets. A comparison between Fig. 6a,b reveals that although the MODIS-based inversion model introduces a systematic overestimation bias, the MODIS-derived RSI effectively distinguishes between high and low SOC regions, demonstrating the stability of spectral indices in practical remote sensing applications. Similar to the inversion results, despite the Landsat-based inversion model and RSI not achieving the highest accuracy among the three datasets, both their mapping outputs are the closest to the reference data. Meanwhile, the Sentinel-derived RSI exhibits an overall overestimation of SOC in the central and western regions of Jilin Province compared to the reference data.

## Discussions

Overall, RSI demonstrates strong potential for accurately identifying and mapping the spatial distribution of topsoil SOC at the provincial scale across multiple satellite datasets, making it a promising tool for broader applications. However, during the experimental process, some limitations and interesting findings emerged, warranting further discussion.

Based on the sensitivity coefficient matrices of the RSI band combinations shown in Fig. 4, the results indicate a strong positive correlation between SOC content and the ratio of shortwave infrared (SWIR) reflectance to reflectance in the visible bands, particularly within the red to near-infrared (NIR) region. Mechanistically, SWIR wavelengths contain absorption features associated with organic functional groups^35,36^, and have consistently been identified as characteristic bands for SOC estimation in proximal sensing studies that rely on laboratory-acquired soil hyperspectral data^37–39^. The positive relationship between SWIR/VNIR reflectance ratios and SOC content arises because VNIR wavelengths respond more strongly to SOC-induced darkening. Soils rich in organic carbon frequently exhibit concave reflectance curves between 0.5 μm and 1.3 μm, whereas soils with low SOC display convex or sigmoidal shapes^40,41^. The SOC-related reduction in reflectance is therefore more pronounced in the VNIR region than in the SWIR region, resulting in higher RSI values corresponding to higher SOC levels.

**Fig. 7:**
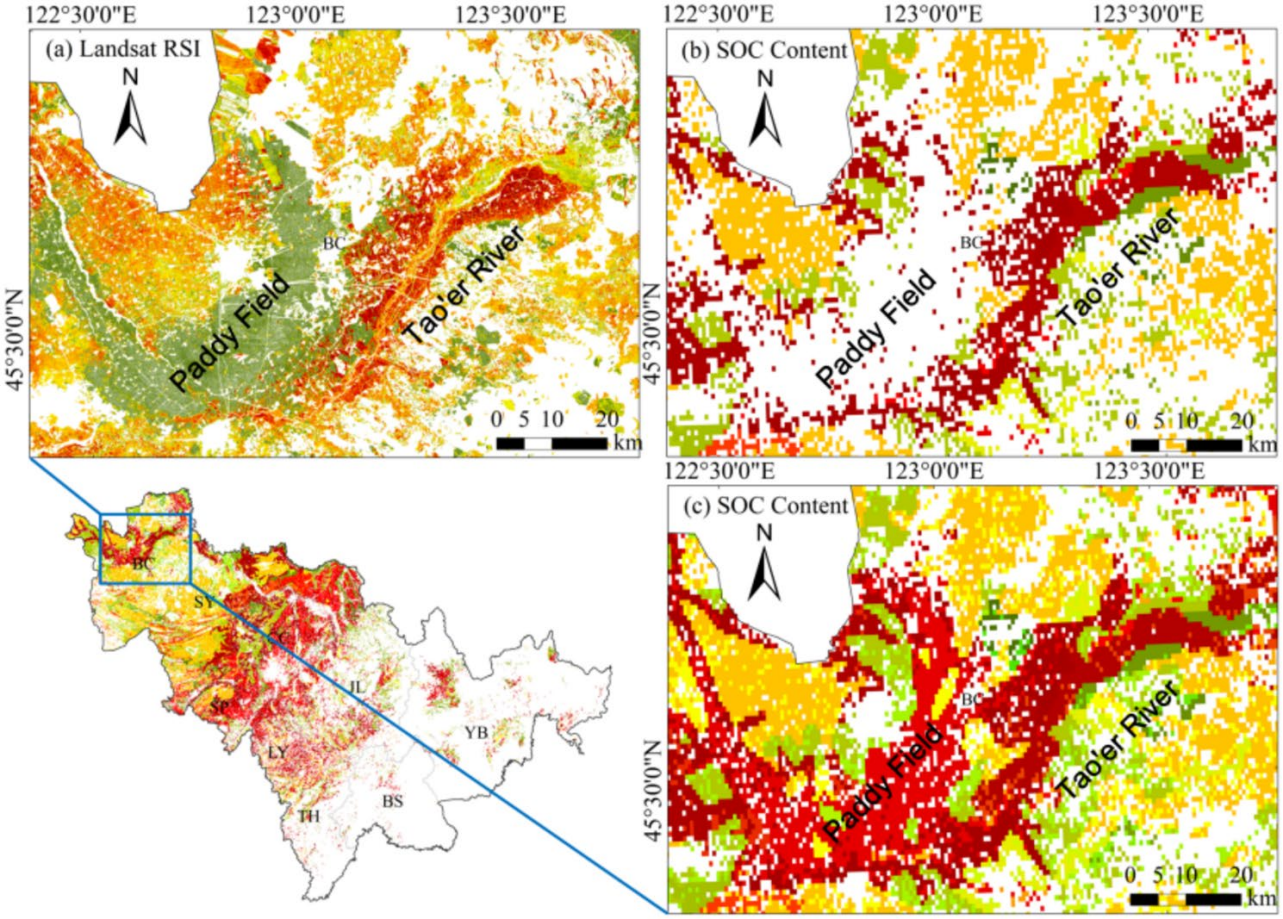
Enlarged mapping results from Fig. 6. (**a**) Landsat RSI mapping result. (**b**) Spatial distribution of observed SOC content excluding paddy fields. (**c**) Spatial distribution of SOC content including paddy fields. Notes: Coordinate system CGCS2000/3° Gauss–Krüger Zone 41; The “Lower” and “Upper” of RSI refer to the 95% confidence interval. This figure was generated in ArcGIS 10.2 software (https://www.esri.com/zh-cn/home).

As shown in Fig. 7, a localized area in the northern part of the study region, specifically in Baicheng, was selected for further examination. Taking Landsat-derived RSI as an example (Fig. 7a) and comparing it with the observed SOC distribution in the region (Fig. 7b,c), it is evident that RSI successfully captures the high SOC spatial distribution in the Tao’ er River floodplain farmland. By referencing the 10-m crop type maps data set, the observed SOC spatial distribution was examined under two conditions: excluding paddy fields (Fig. 7b) and including paddy fields (Fig. 7c). The results indicate that RSI systematically underestimates SOC in paddy field areas. Upon reviewing all results, we found that this underestimation phenomenon is consistent across multiple paddy field regions. This discrepancy is likely caused by uncertain spectral characteristics influenced by land cover variability^31^. The paddy field cultivation system in this region involves straw retention after autumn harvest, followed by tillage and irrigation in spring, resulting in a very limited bare soil exposure window. Consequently, the soil spectral features in satellite imagery are significantly affected by straw residues and soil moisture, leading to reduced RSI accuracy^42^. Therefore, in future RSI applications, its applicability to non-paddy field regions should be prioritized. Additionally, future research will focus on quantifying the effects of soil moisture and straw cover on RSI performance, aiming to parameterize these influences and enhance the general applicability of RSI. Meanwhile, as shown in Fig. 6, the spectral index values calculated from different satellite images for the same region exhibit varying numerical ranges. However, this does not affect the relative description of the spatial distribution of SOC levels using the RSI, similar to what is achieved by other spectral indices such as NDVI. Nevertheless, due to the nature of the ratio-based index, the value range of RSI cannot be easily normalized to a standardized scale (e.g., 0 to 1) like NDVI, which limits its ease of use. Therefore, how to further optimize RSI remains an important direction for future research.

**Fig. 8:**
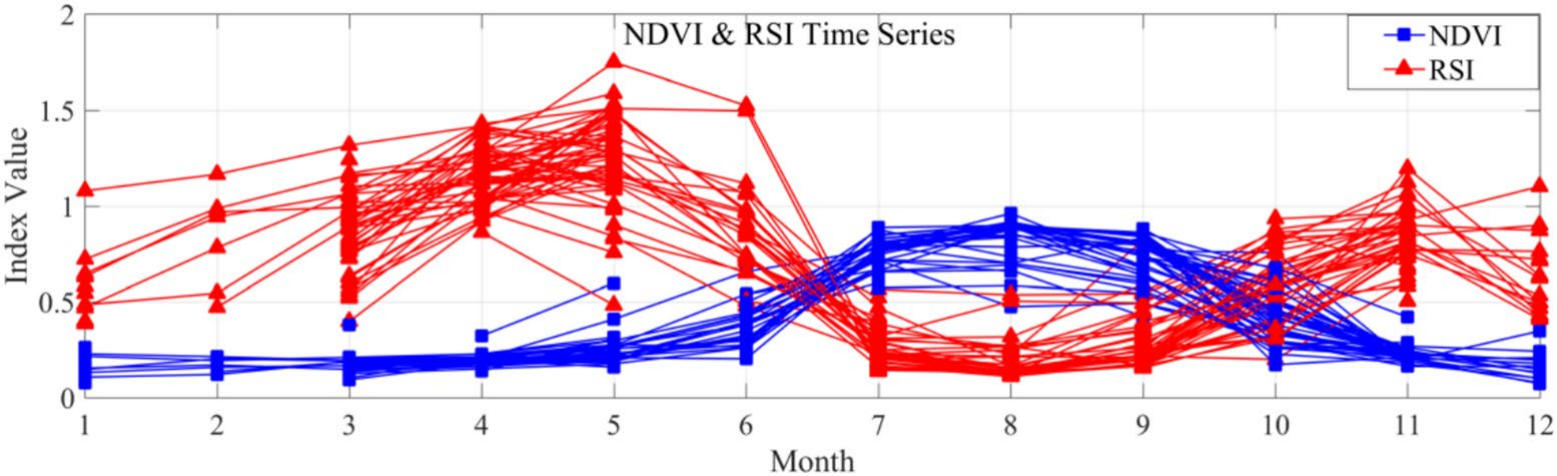
Temporal variation of Landsat-NDVI and Landsat-RSI for sample sites in 2024.

Additionally, another interesting finding emerged during the experiment. Based on the previous results, RSI has been confirmed to exhibit a strong response to SOC, making it an effective spectral index for SOC spatial distribution assessment and mapping. However, beyond this primary function, we also observed that RSI and NDVI exhibit a significant inverse relationship over time. As shown in Fig. 8 using Landsat-RSI as an example, Landsat 8 OLI data from 2024 were used to extract sample site values across the study area. Monthly median NDVI and RSI values were computed to generate a time-series data set spanning January to December. This result suggests that RSI not only enables quantitative assessment of specific soil-related properties but also exhibits an inverse response to vegetation, opposite to NDVI. This indicates that RSI can also serve as a vegetation identification tool—or more precisely, an effective index for detecting bare soil.

Moreover, the impact of sample size in spectral analysis on the generalizability of conclusions is an issue that should be mentioned. Soil spectral characteristics are primarily influenced by chromophores such as organic carbon, clay minerals, water, and iron oxides^10^, The interactions among these components are complex and highly uncertain^11^. Studies have shown that spectral characteristics derived from a limited number of samples may not be representative of spectral features widely applicable across diverse soil systems^15,43^. Given the relatively small sample size in this study, the conclusions from the spectral analysis may carry a degree of uncertainty^44^. Similar studies have also reported variations in accuracy when conducting replication experiments^33^. In addition to uncertainties associated with sample size, the use of satellite images acquired from adjacent years to improve spatial coverage of the study area may introduce additional sources of uncertainty. Although image selection was restricted to the same or comparable growing seasons, subtle interannual differences in surface cover conditions, soil moisture, and atmospheric states may still influence soil spectral responses. These factors are particularly pronounced in cropland environments, where crop residue cover and soil moisture conditions often vary substantially between years. Such temporal inconsistencies may lead to variations in spectral characteristics and, consequently, affect the identification of optimal band combinations. As shown in Fig. 3, both the Landsat ratio index and Sentinel ratio index not only achieved the highest correlation coefficients with SOC at specific band combinations but also exhibited strong correlations when using SWIR bands in combination with NIR and other bands. Although the accuracy and mapping performance of RSI were validated in this study, variations in soil spectral characteristics caused by differences in soil samples may influence the optimal band combinations identified in the analysis. Therefore, to further assess the reproducibility of RSI in other datasets and its applicability across different regions, additional soil samples and more temporally precise synchronized observations should be introduced for further validation.

In summary, the development of RSI enriches the methodological framework of soil remote sensing and demonstrates its applicability through multisource remote-sensing analyses at the provincial scale. The construction of RSI and its multi-temporal validation reveal a declining trend in SOC content across the croplands of Jilin Province over the past seven years, indicating strong potential for future high-frequency monitoring when combined with the advantages of multisource satellite observations. Nevertheless, the preceding discussion highlights that RSI may be affected by SOC underestimation caused by surface cover heterogeneity, as well as by sample-size limitations that could lead to variability in band sensitivity and reduced robustness when applied across regions. Future studies should therefore incorporate normalization or machine-learning approaches to mitigate or parameterize the influences of atmospheric effects, surface cover, and soil moisture on RSI, thereby further improving its performance. Moreover, broader cross-regional validation based on larger sets of ground measurements will be necessary to assess the reproducibility and operational applicability of RSI for SOC monitoring.

## Materials and methods

**Fig. 9:**
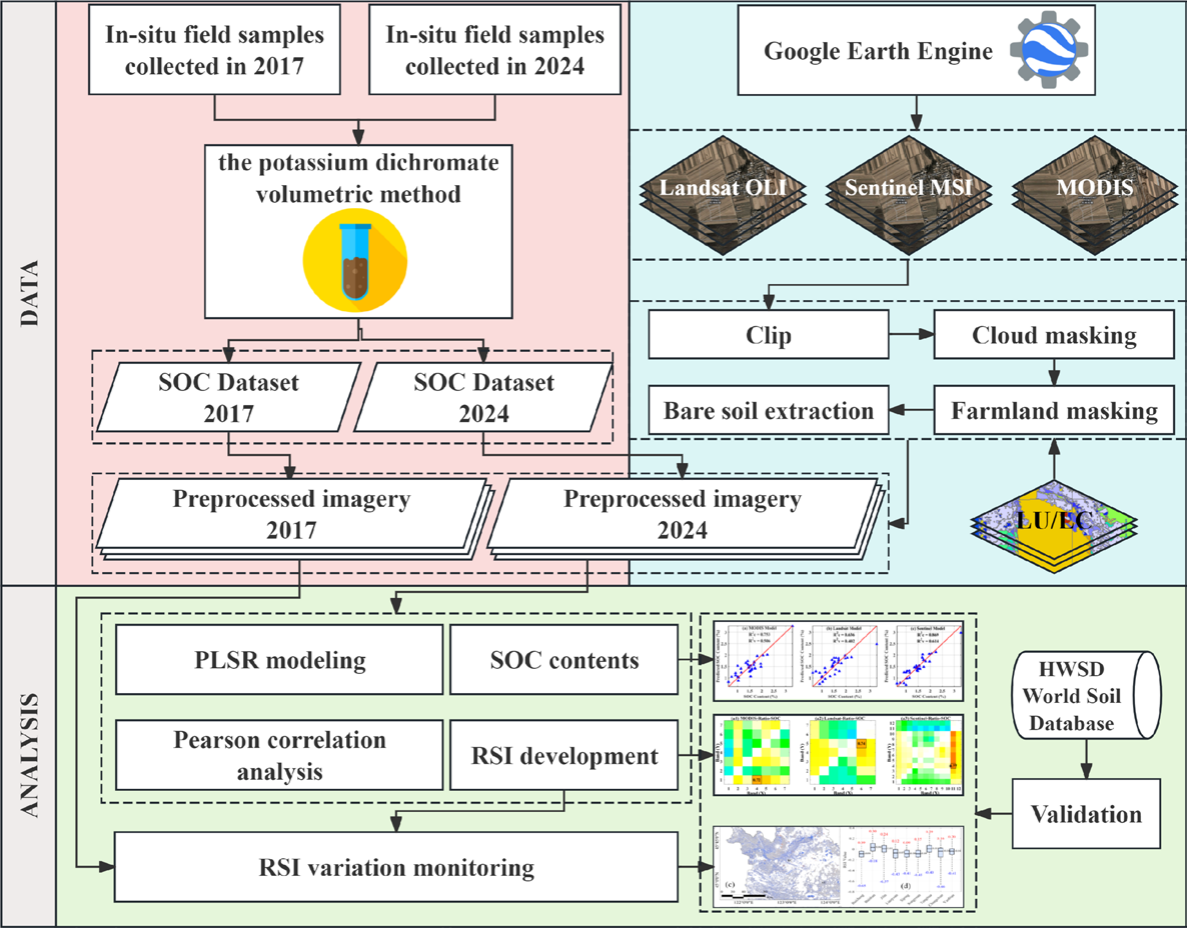
Workflow of the study.

The methodological workflow of this study comprises four major components including data acquisition, preprocessing, spectral analysis and mapping, as shown in Fig. 9.

### Study area

The study area is in Jilin Province, China (40°52′N to 46°18′N, 121°38′E to 131°19′E). It has a temperate continental monsoon climate, transitioning from humid in the southeast to semi-humid and semi-arid in the northwest. The Changbai Mountains in the southeast create a distinct topographical gradient, with higher elevations in the southeast and lower elevations in the northwest. As a result, about 70% of the croplands are concentrated in the central and western regions. Jilin is a major grain-producing region in China, with croplands primarily consisting of dryland fields and paddy fields. The cropping system follows a single annual harvest, with maize and rice as the main crops, growing from May to September^45^.

### In-situ sampling sites

**Fig. 10:**
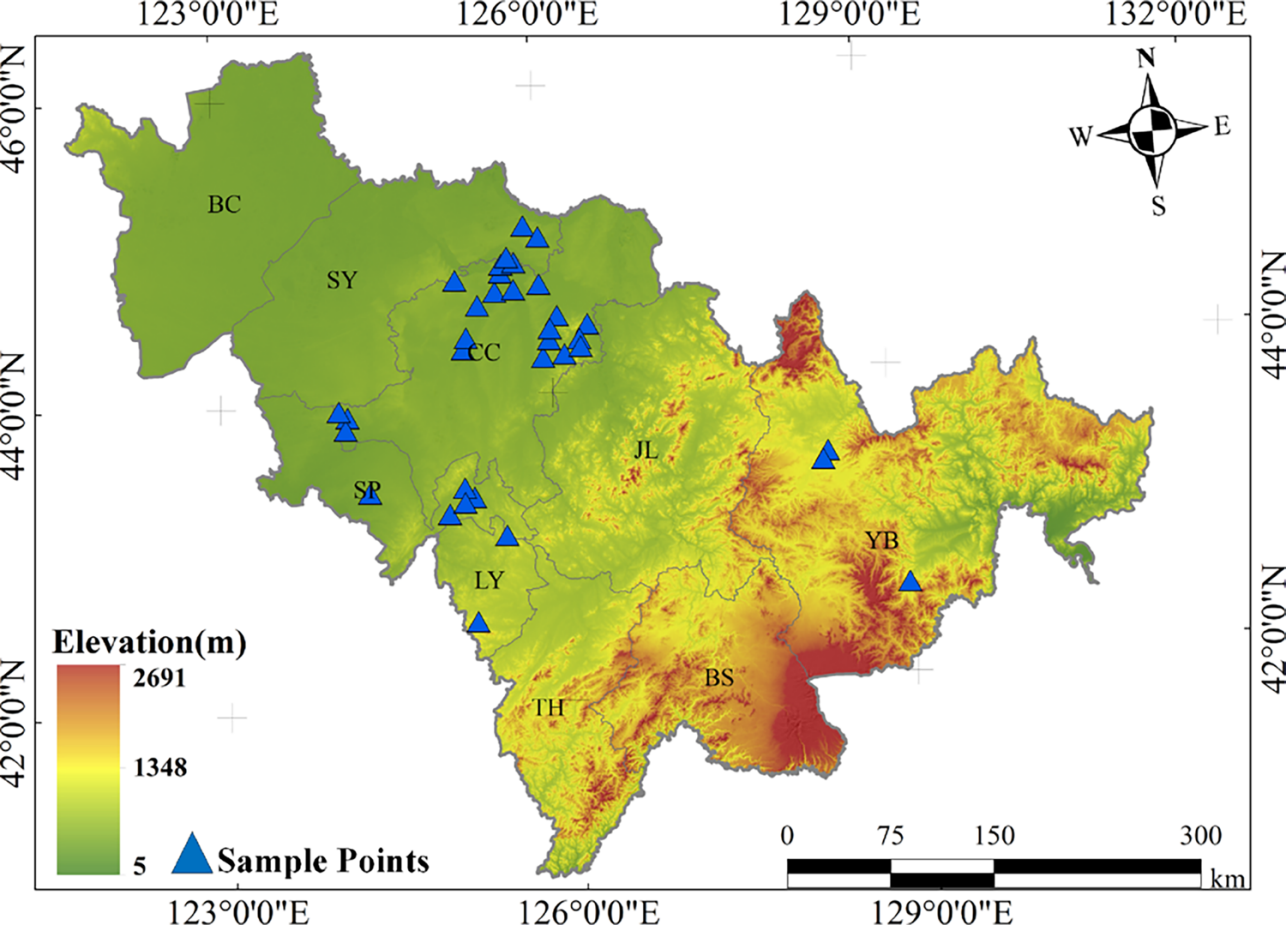
Study area and sample points of the study. This figure was generated in ArcGIS 10.2 software (https://www.esri.com/zh-cn/home).

In 2017 and 2024, during the overpass periods of the Landsat 8 OLI and Sentinel-2 satellites, two rounds of soil sampling were conducted at 68 sites across the study area (Fig. 10), with their coordinates recorded using the WGS84 system. During the 2024 field campaign, several sites sampled in 2017 could not be revisited owing to substantial alterations in local land-cover conditions. These included the conversion of croplands into transportation infrastructure, notable increases in canopy cover from nearby perennial woody vegetation that obscured the original sampling locations, and changes in road accessibility associated with modifications to local transportation infrastructure. As a result, the number of sites available for paired resampling decreased, thereby limiting the sample size for direct interannual comparison. The collected soil samples were air-dried, ground, and sieved through a 0.075 mm mesh in the laboratory for geochemical analysis. SOC content was determined using the potassium dichromate volumetric method^46^. Descriptions of the two datasets are listed in Table 3. The 2024 ground-measured samples, together with the concurrently acquired satellite imagery, served as benchmark data for developing the SOC estimation models and spectral indices, whereas the 2017 samples were used as an independent external dataset for validating the accuracy of SOC change detection.

**Table 3:** Soil data set for this research.

**Year**	**Minimum**	**First quartile**	**Median**	**Third quartile**	**Maximum**	**Mean**	**Standard deviation**	**Coefficient of variation**
2017	1.03	2.09	2.44	2.76	5.33	2.49	0.77	0.31
2024	0.62	1.13	1.49	1.69	3.28	1.48	0.50	0.34

### Remote sensing data acquisition

The study obtained Landsat 8 OLI Collection 2 Level 2 SR, Sentinel-2 MSI SR, and MODIS Terra Surface Reflectance 8-Day Global 500m Surface Reflectance data from Google Earth Engine (GEE) for multi-model calibration and validation (Google LLC, 1600 Amphitheatre Parkway, Mountain View, CA, USA). These datasets are standardized surface reflectance (SR) products for which sensor radiometric calibration, atmospheric correction, and cloud/cloud-shadow QA metadata generation have been uniformly performed by the data providers. Cloud and cloud-shadow removal was conducted using the QA or pixel-QA bands embedded in each image. Because all datasets used in this study are based on consistently standardized SR products and the same cloud-masking procedure was applied across sensors and time periods, comparability among multi-source and multi-temporal datasets is ensured. The preprocessing and verification scripts for all data sources are provided in the Data Availability section at the end of this paper. In the study area, bare soil is typically exposed during the spring plowing period in May each year. However, to minimize uncertainties in surface cover and ensure the acquisition of bare soil pixel spectra, all available imagery during the bare soil period was composited using the GEE workflow. A per-pixel filter was applied to extract potential bare soil pixels based on the following criteria: NDVI < 0.35, NBR2 < 0.125, and BSI > 0.021^47^.This bare soil pixel selection procedure is consistent with the cropland application scenario, spring seasonal characteristics, and Landsat-based SOC estimation cases presented in the study^47^, making it highly relevant and informative for reference. A median composite approach was then used to generate a pixel-level bare soil reflectance image for further spectral analysis. Since Landsat OLI data from 2024 do not fully cover the study area during the bare soil period, supplementary imagery from May 2022 and 2023 was incorporated, considering that SOC remains relatively stable over short periods^48^. For change detection analysis, the 2017 Landsat OLI composite was generated from available imagery in May 2016, 2017, and 2018. Meanwhile, Sentinel-2 MSI and MODIS imagery were exclusively obtained from May 2024.

### Other reference data

The Harmonized World Soil Database (HWSD) Version 1.2 provides global soil physical and chemical attribute data at a spatial resolution of 1 km (FAO). The topsoil organic carbon information within the database can serve as a reference for the spatial distribution of real SOC content when comparing the results of the spectral index calculations and model estimations in this study. The GlobeLand30 land cover dataset (GLC30) is a global scale land cover classification dataset with a 30 m spatial resolution. The GLC30 V2020^49,50^, which has an overall accuracy of 85.72% (Kappa:0.82) (http://www.globallandcover.com), was used in this study to extract the farmland regions. The 10-m crop type maps in Northeast China^51^with an overall accuracy of 95.6% (Kappa: 0.93) (http://www.geodata.cn), were used to distinguish between paddy and dryland areas for further analysis.

### Partial least squares calibration

Partial least squares regression (PLSR) was applied to develop calibration and validation models for SOC content estimation. This method is widely used for handling high-dimensional collinear hyperspectral data in soil property modeling, including SOC estimation^21,52^. In this study, the SIMPLS algorithm in MATLAB (MATLAB 2016b MathWorks, Inc., USA) was used to transform the original spectral variables into orthogonal latent variables^53^, which are linear combinations of the input features. These components were then utilized to establish linear regression relationships with the dependent variables.

### Correlation analysis

The Pearson correlation coefficient was used to calculate the correlation between SOC content and spectral indices. We extracted the reflectance spectra of all bands from any image data at the in-situ sampling locations and iterate through all bands to compute the spectral difference, ratio, and normalized difference combinations for any two bands. The correlation coefficients between any two-band spectral indices and the corresponding SOC content were calculated using the Pearson correlation coefficient and mapped to the corresponding positions in a coefficient matrix. By iterating through all spectral bands, a full-band correlation coefficient matrix was obtained. This matrix provides a quantitative measure of the sensitivity of spectral indices formed by different band combinations to SOC. The results can be visually represented as 3D plots or 2D projections, making this method intuitive, computationally efficient, and easy to implement for spectral sensitivity analysis^33,54^.

### Validation

This study employed the root mean square error of calibration (RMSEc) and cross-validation (RMSEv), along with the coefficients of determination for calibration and validation (R^2^c and R^2^v), to quantify the estimation accuracy. We performed a 10-fold cross-validation, in which the full dataset was randomly partitioned into ten approximately equal subsets. In each iteration, nine subsets were used for model calibration and the remaining one for validation. This process was repeated ten times, and the average RMSEv and R^2^v across all folds were reported. Randomization was performed using a fixed random seed to ensure reproducibility.

## Conclusion

Using Landsat-derived RSI, this study revealed an overall SOC decline of approximately 5.14% across Jilin’s croplands between 2017 and 2024, with the most pronounced reductions occurring in the western plains, patterns that align with previous reports and field-measured SOC data. The RSI defined as the ratio between SWIR and NIR reflectance, provides a concise and interpretable indicator for SOC evaluation. Its effectiveness was consistently validated using MODIS Terra, Landsat OLI, and Sentinel-2 MSI, demonstrating strong generalizability across sensors. Compared with PLSR-based statistical models, RSI shows greater robustness for provincial-scale applications and reliably captures SOC dynamics in multi-temporal monitoring. RSI also exhibited a negative temporal relationship with NDVI, underscoring its potential for bare soil identification. Collectively, these findings highlight RSI as an operational and scalable tool for monitoring SOC change in intensively cultivated regions, offering meaningful support for precision soil management, sustainable agricultural planning, and future efforts to extend SOC monitoring across broader black soil regions.

## Data Availability

The satellite remote sensing data used for spectral index mapping can be accessed via the Google Earth Engine (GEE). And the GEE script is available at: https://code.earthengine.google.com/cfaa76a180574b0b0226783e7e387c76. Additional data related to this paper are available from the corresponding author upon reasonable request.

## References

[1] Wiesmeier, M. et al. Soil organic carbon storage as a key function of soils - a review of drivers and indicators at various scales. *Geoderma* **333**, 149–162 (2019).

[2] Rui, L. I. et al. Soil degradation: a global threat to sustainable use of black soils. *Pedosphere* **35**, 264–279 (2025).

[3] Zhao, Jin et al. For the protection of black soils. *Nat Food* **6**(119), 120 (2025). 10.1038/s43016-025-01126-x 39948395

[4] Schmidt, M. W. et al. Persistence of soil organic matter as an ecosystem property. *Nature* **478**, 49–56 (2011). 21979045 10.1038/nature10386

[5] Cheng, H. et al. Study of loss or gain of soil organic carbon in Da’an region, Jilin Province in China. *J. Geochem. Explor.* **112**, 272–275 (2012).

[6] Gholizadeh, A. et al. Soil organic carbon and texture retrieving and mapping using proximal, airborne and sentinel-2 spectral imaging. *Remote Sens. Environ.* **218**, 89–103 (2018).

[7] Broeg, T. et al. Using local ensemble models and landsat bare soil composites for large-scale soil organic carbon maps in cropland. *Geoderma* **444**, 116850 (2024).

[8] Zhang, L. et al. A CNN-LSTM model for soil organiccarbon content prediction withlong time series of MODIS-basedphenological variables. *Remote Sens.* **14**, 4441 (2022).

[9] Ben-Dor, E. Quantitative remote sensing of soil properties. *Adv. Agron.* **75**, 173–244 (2002).

[10] Clark, R. N. & Roush, T. L. Reflectance spectroscopy: quantitative analysis techniques for remote sensing applications. *J. Geophys. Res.: Solid Earth* **89**(B7), 6329–6340 (1984).

[11] Stenberg, B. et al. Chapter five - visible and near infrared spectroscopy in soil science. *Adv. Agron.* **107**, 163–215 (2010).

[12] Schwartz, G., Eshel, G. & Ben-Dor, E. Reflectance spectroscopy as a tool for monitoring contaminated soils. *Soil Contam.* 6790 (2011).

[13] Rossel, R. V. et al. Visible, near infrared, mid infrared or combined diffuse reflectance spectroscopy for simultaneous assessment of various soil properties. *Geoderma* **131**, 59–75 (2006).

[14] Chabrillat, S. et al. Imaging spectroscopy for soil mapping and monitoring. *Surv. Geophys.* **40**, 361–399 (2019).

[15] Demattê, J. A. et al. Spectral regionalization of tropical soils in the estimation of soil attributes. *Rev. Ciênc. Agron.* **47**, 589–598 (2016).

[16] Bengera, I. & Norris, K. H. Determination of moisture content in soybeans by direct spectrophotometry. *Isr. J. Agric. Res.* **18**, 124–132 (1968).

[17] Morra, M. J., Hall, M. H. & Freeborn, L. L. Carbon and nitrogen analysis of soil fractions using near-infrared reflectance spectroscopy. *Soil Sci. Soc. Am. J.* **55**, 288–291 (1991).

[18] Mccarty, G. W. et al. Mid-Infrared and near-infrared diffuse reflectance spectroscopy for soil carbon measurement. *Soil Sci. Soc. Am. J.* **66**, 640–646 (2002).

[19] Brown, D. J. Using a global VNIR soil-spectral library for local soil characterization and landscape modeling in a 2nd-order Uganda watershed. *Geoderma* **140**, 444–453 (2007).

[20] Wang, S. et al. Using soil library hyperspectral reflectance and machine learning to predict soil organic carbon: assessing potential of airborne and spaceborne optical soil sensing. *Remote Sens. Environ.* **271**, 112914 (2022).

[21] Rossel, R. A. V. & Behrens, T. Using data mining to model and interpret soil diffuse reflectance spectra. *Geoderma* **158**, 46–54 (2010).

[22] Moura-Bueno, J. M. et al. Stratification of a local vis–nir–swir spectral library by homogeneity criteria yields more accurate soil organic carbon predictions. *Geoderma* **337**, 565–581 (2019).

[23] Bellon-Maurel, V. E. & McBratney, A. Near–infrared (NIR) and mid–infrared (MIR) spectroscopic techniques for assessing the amount of carbon stock in soils e critical review and research perspectives. *Soil Biol. Biochem.* **43**, 1398–1410 (2011).

[24] Ward, K. J. et al. A remote sensing adapted approach for soil organic carbon prediction based on the spectrally clustered LUCAS soil database. *Geoderma* **353**, 297–307 (2019).

[25] Rouse, J. W. Monitoring vegetation systems in the great plains with erts. In *Third NASA Earth Resources Technology Satellite Symposium* **1**, 309–3171 (1973).

[26] Jordan, C. F. Derivation of leaf area index from quality of light on the forest floor. *Ecology* **50**, 663–666 (1969).

[27] Richardson, A. J. & Wiegand, C. L. Distinguishing vegetation from soil background information. *Photogramm. Eng. Remote Sens.* **43**, 1541–1552 (1977).

[28] Gitelson, A. A. Wide dynamic range vegetation index for remote quantification of biophysical characteristics of vegetation. *J. Plant Physiol.* **161**, 165–173 (2004). 15022830 10.1078/0176-1617-01176

[29] Tucker, C. J. Red and photographic infrared linear combinations for monitoring vegetation. *Remote Sens. Environ.* **8**, 127–150 (1979).

[30] Sandholt, I., Rasmussen, K. & Andersen, J. A simple interpretation of the surface temperature/vegetation index space for assessment of surface moisture status. *Remote Sens. Environ.* **79**, 213–224 (2002).

[31] Haboudane, D. et al. Integrated narrow-band vegetation indices for prediction of crop chlorophyll content for application to precision agriculture. *Remote Sens. Environ.* **81**, 416–426 (2002).

[32] Krishnan, P. et al. Reflectance technique for predicting soil organic matter. *Soil Sci. Soc. Am. J.* **44**, 1282–1285 (1980).

[33] Jin, X. et al. Remote estimation of soil organic matter content in the Sanjiang Plain, Northest China: The optimal band algorithm versus the GRA-ANN model. *Agric. For. Meteorol.* **218**, 250–260 (2016).

[34] Meng, X. et al. A long-term global Mollisols SOC content prediction framework: Integrating prior knowledge, geographical partitioning, and deep learning models with spatio-temporal validation. *Remote Sens. Environ.* **318**, 114592 (2025).

[35] Gholizadeh, A. et al. Soil organic carbon estimation using VNIR–SWIR spectroscopy: the effect of multiple sensors and scanning conditions. *Soil Tillage Res.* **211**, 105017 (2021).

[36] Bai, Z. et al. Estimation of soil organic carbon using vis-NIR Spectral data and spectral feature bands selection in Southern Xinjiang. *China. Sens.* **22**, 6124 (2022). 10.3390/s22166124 PMC9413329 36015885

[37] Miloš, B. & Bensa, A. Prediction of soil organic carbon using VIS-NIR spectroscopy: application to red mediterranean soils from croatia. *Eurasian J. Soil Sci.* **6**, 365–373 (2017).

[38] Liu, L. et al. Quantitative retrieval of organic soil properties from Visible Near-Infrared Shortwave Infrared (Vis-NIR-SWIR) spectroscopy using fractal-based feature extraction. *Remote Sens.* **8**, 1035 (2016).

[39] Ribeiro, S. G. et al. Soil organic carbon content prediction using soil-reflected spectra: a comparison of two regression methods. *Remote Sens.* **13**, 4752 (2021).

[40] Huete, A. R. & Escadafal, R. Assessment of biophysical soil properties through spectral decomposition techniques. *Remote Sens. Environ.* **35**, 149–159 (1991).

[41] Francos, N., Ogen, Y. & Ben-Dor, E. Spectral assessment of organic matter with different composition using reflectance spectroscopy. *Remote Sens.* **13**, 1549 (2021).

[42] Mulder, V. L. et al. The use of remote sensing in soil and terrain mapping – a review. *Geoderma* **162**, 1–19 (2011).

[43] Stevens, A. et al. Laboratory, field and airborne spectroscopy for monitoring organic carbon content in agricultural soils. *Geoderma* **144**, 395–404 (2007).

[44] Xu, Z. et al. Evaluating the capability of satellite hyperspectral Imager, the ZY1–02D, for topsoil nitrogen content estimation and mapping of farmlands in black soil area. *Remote Sens.* **14**, 1008 (2022).

[45] Liu, Y. et al. Assessment of spatio-temporal variations in vegetation cover in Xinjiang from 1982 to 2013 based on GIMMS-NDVI. *Acta Ecol. Sinica* **36**, 6198–6208 (2016).

[46] DZ/T 0279. 27-2016: Analysis methods for regional geochemical sample-part 27: Determination of organic carbon contents by potassium dichromate volumetric method. http://www.doc88.com/p-7724868306719.html (accessed on 17 January 2024).

[47] Zepp, S. et al. Optimized bare soil compositing for soil organic carbon prediction of topsoil croplands in Bavaria using Landsat. *ISPRS J. Photogramm. Remote Sens.* **202**, 287–302 (2023).

[48] Dou, X. et al. Prediction of soil organic matter using multi-temporal satellite images in the songnen plain. *Geoderma* **356**, 113896 (2019).

[49] Chen, J., Ban, Y. & Li, S. China: open access to earth land–cover map. *Nature* **514**, 434–434 (2014). 10.1038/514434c 25341776

[50] Chen, J. et al. Global land cover mapping at 30 m resolution: a POK–based operational approach. *ISPRS J. Photogramm. Remote Sens.* **103**, 7–27 (2015).

[51] You, N. et al. The 10-m crop type maps in Northeast China during 2017–2019. *Sci. Data* **1**, 41 (2021). 10.1038/s41597-021-00827-9 PMC7854749 33531510

[52] Castaldi, F. et al. Evaluation of the potential of the current and forthcoming multispectral and hyperspectral imagers to estimate soil texture and organic carbon. *Remote Sens. Environ.* **179**, 54–65 (2016).

[53] De, J. S. Simpls: an alternative approach to partial least squares regression. *Chemom. Intell. Lab. Syst.* **18**, 251–263 (1993).

[54] Inoue, Y. et al. Diagnostic mapping of canopy nitrogen content in rice based on hyperspectral measurements. *Remote Sens. Environ.* **126**, 210–221 (2012).

